# Retraction: Correlations of Pituitary Tumor Transforming Gene Expression with Human Pituitary Adenomas: A Meta-Analysis

**DOI:** 10.1371/journal.pone.0279013

**Published:** 2022-12-06

**Authors:** The PLOS ONE Editors

Following the publication of this article [1], similarities were noted between this article and submissions by other research groups, including manuscripts submitted to *PLOS ONE* and [2] which was under review at the same time as [1].

In addition to the above concerns, this article reports the use of “Begger’s funnel plot,” which is not a recognized statistical test.

The first author stated that this article [1] is the work of the listed authors. They stated that the article was submitted using a publicly accessible computer and they do not know how the similarity with the other article [2] occurred. They indicated that the use of the term “Begger’s funnel plot” may have been a spelling mistake. The concerns regarding similarities with other submissions were not fully resolved by the responses.

In light of these concerns which call into question the validity and provenance of the reported results, the *PLOS ONE* Editors retract this article.

JQX and RZW did not agree with the retraction. XHL, BH, YY, KD, MF, BX, WL, and FF either did not respond directly or could not be reached.
